# Revisiting the role of mitochondria in spinal muscular atrophy

**DOI:** 10.1007/s00018-021-03819-5

**Published:** 2021-04-05

**Authors:** Rachel James, Helena Chaytow, Leire M. Ledahawsky, Thomas H. Gillingwater

**Affiliations:** 1 Edinburgh Medical School: Biomedical Sciences, University of Edinburgh, Edinburgh, EH8 9XD UK; 2 Euan MacDonald Centre for Motor Neurone Disease Research, University of Edinburgh, Edinburgh, EH16 4SB UK

**Keywords:** Survival motor neuron, Mitochondrial dysfunction, Mitophagy, Combinatorial therapy, Motor neuron disease, Neurodegenerative disorders

## Abstract

Spinal muscular atrophy (SMA) is an autosomal recessive motor neuron disease of variable clinical severity that is caused by mutations in the survival motor neuron 1 (*SMN1*) gene. Despite its name, SMN is a ubiquitous protein that functions within and outside the nervous system and has multiple cellular roles in transcription, translation, and proteostatic mechanisms. Encouragingly, several SMN-directed therapies have recently reached the clinic, albeit this has highlighted the increasing need to develop combinatorial therapies for SMA to achieve full clinical efficacy. As a subcellular site of dysfunction in SMA, mitochondria represents a relevant target for a combinatorial therapy. Accordingly, we will discuss our current understanding of mitochondrial dysfunction in SMA, highlighting mitochondrial-based pathways that offer further mechanistic insights into the involvement of mitochondria in SMA. This may ultimately facilitate translational development of targeted mitochondrial therapies for SMA. Due to clinical and mechanistic overlaps, such strategies may also benefit other motor neuron diseases and related neurodegenerative disorders.

## Introduction

**Fig. 1:**
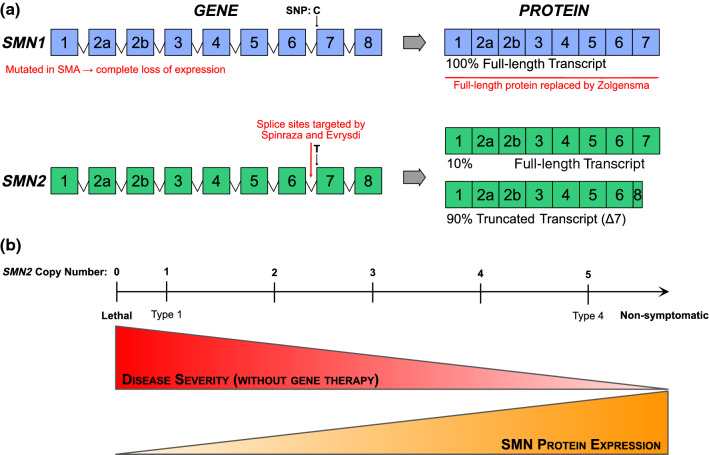
Summary of the genetics of spinal muscular atrophy and their correlation to clinical symptoms. **a** Survival motor neuron (*SMN*)*-1* encodes the SMN protein and is mutated in spinal muscular atrophy (SMA). *SMN2* is a near-identical gene with a single nucleotide polymorphism (SNP) at the beginning of exon 7, leading to exon 7 exclusion in the majority of *SMN2* mRNA transcripts. This truncated protein is quickly degraded. Three currently licenced therapies for SMA (shown in red) aim to increase SMN protein expression, either by introducing an exogenous copy of *SMN1* (Zolgensma^®^) or promoting exon 7 inclusion in *SMN2* transcripts (Spinraza^®^ and Evrysdi^®^), thereby producing full-length SMN. **b** As *SMN2* copy number increases, full-length SMN protein expression increases and is inversely proportional to disease severity. Type 1 patients have one copy of *SMN2*, with copy numbers increasing until Type 4 patients, who have the mildest symptoms

Spinal muscular atrophy (SMA) is a monogenetic motor neuron disease on the verge of being redefined, largely due to notable therapeutic breakthroughs over the last decade. SMA is caused by mutations in the survival motor neuron 1 (*SMN1*) gene, leading to loss of its SMN protein product [1] (Fig. 1a). Due to a complex, highly repeated DNA sequence, the human genome contains an inverted duplication in the *SMN* region of chromosome 5, producing the near-identical duplicate gene, *SMN2*. A single nucleotide polymorphism in exon 7 of *SMN2* leads to removal of exon 7 in 80–90% of transcripts; this truncated transcript is unstable and quickly degraded [2, 3]. *SMN2* is therefore unable to compensate for mutated *SMN1* leading to suboptimal levels of SMN protein. Humans have variable copy numbers of the *SMN2* gene (again due to the repeating nature of this region of chromosome 5), and increased copy numbers of *SMN2* can partially compensate for *SMN1* mutation. This is reflected in time of onset and severity of symptoms in patients [4].

Patients have traditionally been grouped into sub-types of SMA with disease severity and motor milestones influenced by *SMN2* copy number [5] (Fig. 1b). Type 0 patients have one copy of *SMN2*, with prenatal motor weakness, paralysis, and death weeks after birth. Type 1 patients are more common, with two copies of *SMN2* and obvious motor symptoms within the first few months of life as they do not develop head control. Untreated, Type 1 patients have a life expectancy of around 2 years. Patients with three copies of *SMN2* are characterised as Type 2, with symptom onset between 6 months and 2 years and development of motor skills such as sitting and possibly standing. Type 3 patients develop motor symptoms after 18 months old, have three or four copies of *SMN2*, and are likely to reach the motor milestone of unassisted walking and have a normal lifespan [6]. These SMA patient sub-types are based on the natural history of the disease. However, we have reached a new therapeutic era for SMA, with the approval and licencing of three genetic therapies: Spinraza^®^ is an antisense oligonucleotide that modulates splicing of *SMN2* to increase SMN production [7]; Zolgensma^®^ is a viral vector that introduces a second copy of *SMN1* to boost SMN expression [8]; and Evrysdi^®^ is a small molecule that also alters *SMN2* splicing [9]. Treatment with any one of these therapies significantly extends the time to invasive respiratory ventilation and life expectancy for many patients with SMA (most trials to date have been conducted in Type 1 patients). However, emerging evidence from patients treated with these “SMN-replacement” therapies reveals an ongoing loss of function and continued presence of neuromuscular symptoms [10]. Children treated earlier, before motor symptoms develop, have the highest chance of achieving successive motor milestones, while children who are treated at a later disease time point continue to be at high risk of needing supported ventilation [11] and enteral feeding [12]. The effect of these therapies in adults with Types 2/3 SMA is even less clear, not least because of the heterogeneity of clinical symptoms and small patient group sizes. Nevertheless, there is some evidence of stabilisation or even improvement in motor symptoms in adult patients [13]. Thus, there is a growing body of evidence indicating that replacing SMN protein levels in patients can only go part-way to relieving symptoms due to affected tissues being too far down the degeneration pathway [14]. SMN-independent therapies are therefore needed to act in concert with SMN-replacement and further benefit patients with SMA (e.g., “SMN + therapies” [15]).

As a multi-systemic disorder, SMA affects many cell types since SMN protein is ubiquitously expressed throughout the body [16]. Undoubtedly though, motor neurons and muscle cells are a predominant site of cellular pathology. Neurons and muscle cells are in turn two of the most energy-dependent cells within the body; therefore, as mitochondria are primarily known for their role in energy and metabolic pathways, it is not hard to envision their involvement in SMA based upon this role. Yet mitochondria are no longer viewed solely as the ‘powerhouse’ of the cell and are implicated in many other aspects of cellular function that intersect with the aetiology of SMA and known roles of the SMN protein. Initially, SMN protein was reported to function in transcriptional processes but is now known to also play roles in protein translation and related proteostatic mechanisms such as autophagy and ubiquitination [17]. Dissecting these varied functions of SMN across a disease of heterogenous expression has been made possible through the development of animal models of varying severity (e.g., severe, intermediate, and mild mouse models, reflecting Types 1, 2, and 3/4 in patients, respectively) [18, 19]. By providing an overview of the mitochondrial dysfunction in SMA, our intention is to guide molecular mechanistic understanding and aid translational targeting of mitochondria, which offer many routes for complementary therapeutic interventions to current SMN-replacement therapies.

## Mitochondrial dysfunction in SMA

### Mitochondrial respiration and oxidative phosphorylation

Mitochondrial respiration refers to the set of metabolic reactions and processes that require oxygen to generate ATP. Of this process, oxidative phosphorylation mediated via the electron transport chain (ETC; complexes I–V) is the final step. Unsurprisingly, energetic disturbances suggestive of underlying mitochondrial dysfunction are present in muscle biopsies from patients with SMA (Table 1). Overall, SMA muscle pathology is associated with a decrease in mitochondrial respiration, reduced activity in oxidative phosphorylation enzymes, and a concomitant reduction in expression of both nuclear-encoded and mitochondrial-encoded subunits of the oxidative phosphorylation machinery [20–24]. This energetic disturbance has since been corroborated in various animal and cell culture models [25–31]. Interestingly, SMA patient-derived myoblasts and differentiated myotubes showed an intrinsic energy deficit characterised by increased reliance on mitochondrial ATP production [29], that may be borne out in patients with Type 3 SMA [32]. Taken together, these studies reveal a deficit in oxidative phosphorylation activity in tissue and cells relevant to SMA pathology. Moreover, these deficits are evident across the clinical spectrum of SMA and are not solely a feature of the more severe (i.e., Type 1) phenotype.

**Table 1:** Oxidative phosphorylation in spinal muscular atrophy

SMA type/model	Respiration rate	Cellular ATP levels	Enzyme activity	Enzyme	Transcript expression	Transcripts	Protein expression	Proteins	References
Patient skeletal muscle fibres: vastus lateralis of quadriceps (SMA type unknown; mean age 5.8 years)	Reduced		Reduced	Complex I; II–III; IV; PDHC; citrate synthase					[20]
Patient skeletal muscle (Types 1–3)			Reduced	Complex II; IV; citrate synthase			Reduced	MT-CO1; COX4	[21]
Patient muscle (muscle group and SMA type unknown; mean age 3.1 years)			Reduced	Complex I; II; III; I–III; II–III; IV; V; citrate synthase					[22]
Patient quadriceps femoralis muscle (Types 1 and 3)					Reduced (Type 1)	NDUFB1; CYC1; SLC25A4			[23]
Patient quadriceps/paraspinal muscle (Types 1–3)			Reduced	Complex I; II; II–III; IV; citrate synthase	Reduced	MT-ND1; SDHA; MT-CO1; MT-CO2; COX4I1; MT-ATP6	Reduced	SDHA; MT-CO1; MT-CO2; COX4I1	[24]
Patient-derived iPSC-MNs/astrocytes	No change (astrocytes)						No change	Complex II, IV	[27]
Type 1 patient-derived myoblasts/myotubes	Reduced (myoblasts)	No change (at baseline)							[29]
Spinal cord from Taiwanese mice (P5, P8)^a^		Reduced							[28]
Heart from Taiwanese mice (P1)^a^							Increased	MT-ATP6	[30]
Spinal MNs from Hung–Li SMA mice (P9)^b^ Cultured MNs from SMN∆7 mice (E12.5)^b^	Reduced				Reduced				[26]
Flexor tibialis and extensor plantaris (fast-twitch) and extensor soleus (slow-twitch) muscles from Smn∆7/∆7; huSMN2+/+mice^c^			Reduced (fast-twitch) No change	Complex I; II; IV; citrate synthase			No change	NDUFB8; SDHB; UQCRC2; MT-CO1; ATP5A	[31]
Smn morphant zebrafish embryos	Reduced						Reduced	ATP5A	[28]

All five complexes of the oxidative phosphorylation system show alterations in enzyme activity. Expression changes are found in transcripts and proteins encoded by both the nuclear and mitochondrial (denoted by ‘MT-’) genomes across all five complexes: MT-ND1, NDUFB1, and NDUFB8 (complex I); SDHA and SDHB (complex II); CYC1 and UQCRC2 (complex III); MT-CO1, MT-CO2, COX4, and COX4I1 (complex IV); ATP-5A, and MT-ATP6, and SLC25A4 (complex V). Mouse models of SMA are defined as severe^a^, intermediate^b^, or mild^c^ (reflecting SMA Types 1, 2, and 3/4, respectively). *iPSC* induced pluripotent stem cell, *MNs* motor neurons, *PDHC* pyruvate dehydrogenase complex, *SMA* spinal muscular atrophy, *SMN* survival motor neuron

### Reactive oxygen species and oxidative stress

As by-products of oxidative phosphorylation, as well as other mitochondrial and cellular biochemical reactions, reactive oxygen species (ROS) is an umbrella term for a group of molecular species. ROS are well-known for their pathological role in oxidative processes and resultant oxidative stress, but also play a physiological role in cell signalling and homeostasis [33]. Although neither ROS production nor oxidative stress are exclusively mitochondrial functions, both are underpinned by mitochondria and are a classic example of the double-edged role that mitochondria often play in cellular life. Based upon the oxidative phosphorylation defects in SMA, it follows that there is also evidence of altered ROS levels and oxidative stress in post-mortem patient material [34] and models of SMA [25, 26, 30, 35–37] (Table 2). Overall, the main finding has been of increased ROS and oxidative stress in SMA pathology, with any exceptions indicating that tissue/cell-type differences and the type of ROS examined are important. Relevantly, 4-hydroxynonenal (4-HNE) is generated primarily within mitochondria and is a marker of lipid peroxidation [38]. Although not exhaustively examined, 4-HNE is the only oxidative stress marker so far that has been associated with motor neuron pathology [27, 34, 35].

**Table 2:** Reactive oxygen species and oxidative stress pathology in spinal muscular atrophy

SMA type/model	ROS/Oxidative stress marker	ROS/Oxidative stress production	SMN splicing	SMN complex	Rescue	References
Patient spinal cord and brain tissue (Werdnig–Hoffman disease; Type 1)	4-HNE 8HOdG 3-Nitrotyrosine	Increased (MNs) No change (MNs) No change (MNs/brain)				[34]
Type 3 patient-derived spinal MNs	MitoSOX H_2_DCFDA ATF-6	Increased Increased No change			Edaravone	[37]
Patient-derived iPSC-MNs/astrocytes	DHE	Reduced				[27]
Paraquat-treated neuronal and non-neuronal cell lines/patient-derived fibroblasts			Altered		ASO (ISS-N1)	[40]
Stable SMN knockdown in hESC-derived MNs	MitoSOX	Increased				[36]
Paraquat-treated SH-SY5Y human neuroblastoma cells			Altered			[39]
β-Lapachone-treated HeLa cells				Modulated (via crosslinking)		[42]
Smn siRNA knockdown in NSC-34 cells	H_2_DCFDA	Increased				[25]
Cultured MNs from Smn∆7 mice (E12.5)^b^	Mito-roGFP	Increased				[26]
Spinal cord from Smn∆7 mice (P4–8)^b^	8HOdG	No change				[27]
Heart from Smn∆7 mice (P9)^b^	3-Nitrotyrosine	Increased			Adenoviral-SMN	[35]
Paraquat-treated heterozygous Taiwanese mice (6–8 weeks; various tissues)^a^			Altered			[41]

MitoSOX is a commercially available dye used to measure mitochondrial production of superoxide. Mito-roGFP measures the mitochondrial redox state using redox-sensitive GFP (roGFP) that is targeted to the mitochondrial matrix. Mouse models of SMA are defined as severe^a^, intermediate^b^, or mild (reflecting SMA Types 1, 2, and 3/4, respectively). *ASO (ISS-N1)* antisense oligonucleotide targeting the SMN2 intronic splicing silencer N1, *ATF-6* activating transcription factor 6, *DHE* dihydroethidium, *H2DCFDA* 2′,7′-dichlorodihydrofluorescein diacetate, *hESC* human embryonic stem cell, *4-HNE* 4-hydroxynonenal, *8HOdG* 8-oxo-2′-deoxyguanosine, *MNs* motor neurons, *ROS* reactive oxygen species, *SMA* spinal muscular atrophy, *SMN* survival motor neuron

Mechanistically, *SMN1* and *SMN2* splicing [39–41] and activity of the SMN complex [42] can be regulated by ROS in oxidative stress-inducing conditions (Table 2). This suggests that increased ROS may be pathological in SMA through disruption of physiological ROS functions. Concurrently, oxidative stress induced by paraquat (a compound that increases mitochondrial ROS production [43]) reduces SMN protein levels [40, 41]. Therefore, along with reduced levels of SMN protein due to *SMN* mutation, increased mitochondrial ROS production may exacerbate and further reduce SMN protein levels. Whether reduced SMN levels in patients with SMA directly affects mitochondrial ROS-generating processes as shown in cell studies [26, 36, 37], or whether mitochondrial ROS production and oxidative stress are increased due to an external factor (that may also be SMA-related) is not yet clear. To address this question, it is pertinent to consider other aspects of SMA pathology that may be involved in producing an oxidative environment, specifically, the involvement of hypoxia.

Mitochondria are the main oxygen consumers of the cell and are critically dependent on oxygen availability for mediating their canonical function of oxidative phosphorylation. Consequently, they are implicitly involved in oxygen-sensing, and in turn, hypoxic cellular pathways, which interlinks with their role in intermediary metabolism [44]. Hypoxic changes are present in neuronal and non-neuronal tissues of SMA model mice at an early symptomatic time point [45, 46]. Like oxidative stress, hypoxia is mechanistically associated with *SMN2* splicing, promoting exon 7 skipping through heterogeneous nuclear ribonucleoprotein (hnRNP)-A1 (a negative regulatory splicing factor of *SMN2* exon 7) in a severe mouse model of SMA [47]. Therapeutically, hnRNPA1 binding is blocked by Spinraza^®^, which promotes exon 7 inclusion and production of full-length SMN protein [15]. Hypoxic pathways are also triggered at the molecular level by ROS production and hypoxia-inducible factors (HIFs), an oxygen-regulated family of transcription factors that modify glycolytic capacity and are reported sensors of mitochondrial health [48]. Under normal oxygen conditions, HIF1α protein is rapidly degraded by ubiquitination processes involving the SMN-relevant proteins, ubiquitin-like modifier activating enzyme 1 (UBA1) [49, 50] and ubiquitin carboxyl-terminal hydrolase L1 [51, 52]. During hypoxia, increased mitochondrial ROS stabilises HIF1α protein, leading to further activation of hypoxic signalling pathways. Due to functional changes in SMN-related ubiquitination, basal degradation of HIF1α may be altered in SMA with increased mitochondrial ROS production further exacerbating this process. These potential hypoxic interactions between SMN and mitochondria have not been fully explored. Yet hypoxia offers a feasible environmental factor for influencing development of SMA, and unarguably, is a modifying influence on mitochondrial function.

### Mitochondrial metabolism

Mitochondria are integral to metabolic pathways, both in terms of using end products as sources of electrons for the electron transport chain and providing ATP for further metabolic processes. Metabolism in turn is a critical link between molecular pathology and clinical care, since changes in nutritional care of patients can have direct impacts on metabolic function and vice versa. Metabolic dysfunction is an understudied area of research in SMA, but the few studies that have investigated this have found distinct perturbations in various kinds of metabolism across patient groups. Glucose, amino acids, and lipids can all be metabolised to provide substrates for ATP generation in mitochondria [53]. Glucose metabolism is dysregulated in both patients and models of SMA. Mild and intermediate mouse models of SMA show glucose intolerance that increases with age as well as hyperglucagonemia, increased insulin sensitivity, and fasting hyperglycaemia [54, 55]. These metabolic phenotypes are accompanied by morphological changes in the pancreas, with pancreatic islet cells showing an increased proportion of glucagon-producing cells (α cells) and a decreased proportion of insulin-producing cells (β cells). This change in islet cell composition was also found in patients with Type 1 SMA [54]. Patients with SMA are reported to have increased visceral fat mass, and children with Type 2 SMA and obesity are at higher risk for insulin resistance [56].

There is also evidence of amino acid metabolism dysregulation, again in both clinical and pre-clinical SMA research. Branched-chain amino acid (BCAA) pathways showed major changes in expression at early and symptomatic time points in a severe SMA mouse model, notably with reduced BCAA transaminase 2 (*Bcat2*) expression as a major catabolic enzyme of BCAAs [57]. This dysregulation of amino acid metabolism was also found in patients with Type 2 SMA [56]. Dyslipidemia and fatty deposits were found in the liver post-mortem in a cohort of patients with Type 2 SMA, which is mimicked in an intermediate mouse model [58]. This study also reported global dysregulation of fat and lipid metabolism. In the era of SMN-replacement therapies, these secondary characteristics of SMA are likely to become more pressing, both in terms of combinatorial therapy development and long-term disease management. At the cellular level, the ability to effectively switch between energetic fuels in response to fluctuating environmental conditions (such as during development or neuronal signalling) is dependent on the inherent metabolic flexibility of mitochondria, which is mediated by their ability to change shape [59, 60]. Whether dysregulation in all three mitochondrial fuels in SMA pathology reflects loss of this adaptative ability in mitochondria remains to be determined.

### Apoptosis

SMA pathology is primarily defined by motor neuron loss in the spinal cord, which reasonably may occur through apoptosis as a cell fate mechanism. Mitochondria are well-known as key activators of the intrinsic pathway of apoptosis [61], which involves a combination of pro- and anti-apoptotic proteins, including Bcl-2 family members, caspase family members, cytochrome C, and p53. In support of a role for apoptosis in SMA, several studies have shown altered levels of these proteins in patients and models of SMA [62]. Further involvement of SMN protein at the molecular level can be inferred through SMN interactions with known apoptotic proteins. Recombinant SMN directly interacts with the anti-apoptopic and outer mitochondrial membrane (OMM) protein, Bcl-2, to exert a synergistic effect against apoptosis in cell culture studies [63, 64]. Although the physiological significance of this interaction has not been confirmed [65], there is ample evidence implicating Bcl-2 and other family members across the spectrum of SMA pathology in patient tissue [66], animal models [67], and cell culture models [68, 69]. Another SMN interaction of interest to apoptosis is the pro-apoptotic protein, p53, which in turn regulates the Bcl-2 family of proteins [70]. P53 is a transcription factor involved in cell stress and can both induce apoptosis (nuclear p53) and repress autophagy (cytosolic p53), which in part is dependent on p53 translocation to external (OMM) and internal (matrix) mitochondrial locations [71]. Nuclear p53 activity is selectively upregulated in disease-vulnerable spinal motor neurons in an intermediate mouse model of SMA [72], while direct interaction between SMN and nuclear p53 correlates with SMA disease severity in patient-derived fibroblasts [73]. Zinc-finger protein 1 (ZPR1) is another apoptosis-inducing protein and SMN interactor [74, 75]. ZPR1 expression levels are reduced in patients with SMA and may play a role in disease severity [76]. Corroboratively, ZPR1 was identified as a potential modifier of SMA pathology in mild and severe mouse models of SMA, with alternating effects on apoptosis that are dependent on ZPR1 expression levels [77]. Finally, the gene for neuronal apoptosis inhibitory protein (or NLR family apoptosis inhibitor protein; NAIP) is located within the genomic region of chromosome 5 that encompasses *SMN1*. *NAIP* is not the causative gene for SMA but *NAIP* mutations are suggested to modify disease severity in patients with SMA [78, 79], albeit their clinical significance is unclear [4]. Nevertheless, as NAIP is regulated by release of caspases 3 and 9 during apoptosis [80], it is reasonable that *NAIP* may play a modifying role in apoptosis of SMA motor neurons [81, 82]. Aside from apoptosis, NAIP is most studied for its role in another mitochondrial pathway, that of activation of the innate immune system [83]. Thus, clarifying the mitochondrial role of Bcl-2, p53, ZPR1, and NAIP in apoptosis may shed further light on cell death mechanisms in SMN disease-vulnerable motor neurons.

### Mitochondrial trafficking

Mitochondria constantly move around the cell to meet local metabolic and energetic demands, and are trafficked along the cellular cytoskeleton [84]. Cytoskeletal and trafficking processes have previously been implicated in SMA [17], suggesting that reasonably, SMN may also play a role in mitochondrial trafficking. Accordingly, *smn-1* was identified from a genetic screen in *C. elegans* to detect components of abnormal mitochondrial localisation [85]. Through interactions with actin-related protein 2 (ARX-2), *smn-1* was subsequently shown to influence mitochondrial localisation and distribution in muscle myofilaments. Patient-derived cell culture models of SMA show motor neuron-specific reductions in mitochondrial movement along axons [26, 86, 87] (Table 3). Reduced mitochondrial trafficking in SMA cell models may therefore reflect dissociation of SMN interactions between ARX-2 and actin filaments. Alternatively, tubulin-related mitochondrial trafficking is commonly implicated in other forms of SMA [88, 89] and motor neuron diseases [90], while intermediate filament aggregation disrupts mitochondrial movement in giant axonal neuropathy, a related neurological disorder [91]. Determining whether these cytoskeletal alterations in mitochondrial trafficking across disease classifications share SMN-related molecular disturbances may enable utilisation of shared modelling and therapeutic approaches.

**Table 3:** Mitochondrial abnormalities in spinal muscular atrophy

SMA type/model	Trafficking	Distribution	mtDNA content	Number	MMP	Morphology	Size	Rescue	References
Patient skeletal muscle (Types 1–3)			Reduced						[21]
Patient quadriceps/paraspinal muscle (Types 1–3)			Reduced (correlated with disease severity)						[24]
Type 1 patient-derived MNs from iPSCs/hESCs	Reduced			Reduced	Reduced		Reduced	*N*-Acetylcysteine (antioxidant)	[86]
Type 1 patient-derived iPSC-MNs	Reduced			Reduced			Reduced	Z-FA-FMK (protease inhibitor)	[87]
Type 1 patient-derived myoblasts/myotubes			No change						[29]
Smn siRNA knockdown in NSC-34 cells					Increased				[25]
Diaphragm from SMN∆7 mice (P14)^b^				No change (presynaptic)			Reduced (presynaptic)		[95]
Tibialis anterior muscle from SMN∆7 mice (P13)^b^				Reduced (presynaptic)		Normal (presynaptic)			[97]
Tibialis anterior muscle from SMN∆7 mice (P14)^b^		Normal (presynaptic)		Reduced (presynaptic)					[96]
Cardiomyocytes from SMN∆7 mice (P14)^b^						Degenerative	Swollen		[105]
Spinal cord (P3, P9) and cultured MNs (E12.5) from SMN∆7 mice^b^	Reduced				Reduced	Fragmented/abnormal	Reduced		[26]
Diaphragm/soleus from Taiwanese mice (P4)^a^						Fragmented/abnormal (diaphragm)	Swollen (diaphragm)	*SMN2* splicing	[106]
Intercostal muscles from Taiwanese mice (P4)^a^						Normal	Normal		[108]
Cervical spinal cord from Taiwanese mice (P3)^a^						Abnormal	Reduced		[107]
Tibialis anterior muscle from SMN2B mice (P21)^b^		In vacuoles							[103]
*C. elegans* smn-1 mutant body wall muscle		Abnormal				Abnormal			[85]

Mouse models of SMA are defined as severe^a^, intermediate^b^, or mild (reflecting SMA Types 1, 2, and 3/4, respectively). *hESCs* human embryonic stem cells, iPSCs induced pluripotent stem cell, *MMP* mitochondrial membrane potential, *MNs* motor neurons, *mtDNA* mitochondrial DNA, *SMA* spinal muscular atrophy, *SMN* survival motor neuron

### Mitochondrial quality control mechanisms

#### Mitochondrial dynamics

Overlapping with a role in mitochondrial trafficking, the cytoskeleton is also involved in mitochondrial dynamics, which describes the intertwined fission and fusion capabilities of mitochondria [92]. Mitochondrial fission is a means of both mitochondrial replication and removal of damaged or dysfunctional mitochondria (i.e., mitophagy). Mitochondrial fusion enables the exchange of metabolites and other molecules (including mitochondrial DNA [mtDNA]) among mitochondria and leads to generation of the interconnected mitochondrial networks that are present in many cell types, such as neurons and muscle. Related to mitochondrial dynamics are mitochondrial biogenesis (generation of new mitochondria from pre-existing mitochondria) and mitophagy [93]. Taken together, these interrelated functions are viewed as quality control mechanisms to maintain a healthy mitochondrial population and optimise the energetic and metabolic output of the cell. Mitochondrial dynamics have already been implicated in the pathogenesis of neurodegenerative diseases [94], but have not yet become an area of active research for SMA. Despite this, there are indications that mitochondrial dynamics may be disrupted in *smn-1* mutant worms, with disruption of the typical elongated mitochondrial network in the belly wall muscle [85]. This phenotype is supported by SMA cell models [26, 86, 87] showing reduced mitochondrial length, a concomitant increase in fragmentation, and reductions in fusion transcripts (Table 3). Overall, these changes are suggestive of impaired mitochondrial fusion in SMA.

#### Mitochondrial biogenesis and mitophagy

Mitochondrial biogenesis can be inferred through regulation of relevant transcriptional pathways, or more directly shown by the amount of mtDNA and/or number of mitochondria. There is evidence that all of these processes are altered in SMA pathology [21, 24, 26, 86, 87, 95–97]. In particular, reduced mtDNA content has been reported in patient muscle tissue [21, 24] that partially tracks with the clinical phenotype [23]. Reduced mitochondrial number and/or density are found in mouse [95–97] and cell culture [86, 87] models of SMA (Table 3). Importantly, there are muscle-specific differences that correlate with the disease phenotype: fewer mitochondria are detected in the disease-vulnerable tibialis anterior muscle of a severe SMA mouse model [96, 97], while no change was reported in the diaphragm at a similar postnatal stage [95]. Together these findings suggest there are less mitochondria in pathologically relevant cell types. However, it is not known how reduction of SMN protein and function is involved. Interestingly, there are clinical reports of mitochondrial phenocopies of SMA [98], defined as a disease that phenotypically resembles SMN-associated SMA but without *SMN1* gene mutations. These phenocopies also show mtDNA depletion [99–102] and may prove useful for mechanistic understanding of the relationship between mtDNA content and SMA development.

Due to the reduced energetic capability of mitochondria in SMA, mitophagy should be activated as a cellular response to remove dysfunctional mitochondria. Vacuoles containing mitochondria were reported in an intermediate mouse model of SMA, and not in control mice [103], suggesting that mitophagy is activated in SMA. Mitophagy can be indirectly inferred by depolarisation of the mitochondrial membrane potential (MMP), which reflects the functional status and viability of mitochondria [104]. This has been examined in cell models of SMA [25, 26, 86] (Table 3), with stable knockdown of SMN levels associated with a depolarised MMP. This suggests that the mitochondrial population is functionally suboptimal and a mitophagic response should be induced. Further investigation of this process is needed to determine how altered SMN autophagic function may be involved, possibly preventing efficient removal of dysfunctional mitochondria.

### Mitochondrial morphology and size

Mitochondrial morphology and size are remarkably heterogenous among cell types and are regulated in part by mitochondrial dynamics and the cytoskeleton. Together they impact upon the mitochondrion’s energetic and metabolic output. Morphological abnormalities of mitochondria are evident in animal models of SMA [26, 85, 105–107] (Table 3), including alterations in cristae (the mitochondrial location of the ETC) at a pre-symptomatic stage of disease [26]. In addition, both larger [105, 106, 108] and smaller [26, 95, 107] mitochondria have been reported in animal models of SMA (Table 3). Swollen or enlarged mitochondria may represent degenerative mechanisms acting downstream of reduced SMN protein levels. While smaller mitochondria may reflect a physiological phenotype of SMA pathology: indeed smaller mitochondria are associated with reduced SMN levels in cell culture models of SMA [86, 87].

### Mitochondrial import of proteins

Due to the small number of proteins encoded by the mitochondrial genome (13 of > 1000 mitochondrial proteins in humans [109]), mitochondria must import the rest of the proteins and other macromolecules needed for its function from the cytosol. Import occurs through protein complexes located within the outer and inner mitochondrial membranes, namely translocases of the outer membrane (TOM) and inner membrane (TIM) [110]. Mitochondrial import has not been directly investigated in SMA research, but there is tentative evidence that this process could be affected by SMA pathology. Reduced expression of mitochondrial import proteins has been shown in patient muscle biopsies [24] and patient-derived spinal motor neurons [111], albeit not in a mild mouse model of SMA [31]. Further, voltage-dependent anion-selective channel 1 (VDAC1) is an OMM import protein that interacts with stasimon, a protein involved in synaptic transmission and already implicated in SMN pathways [112]. VDAC1–stasimon interactions occur at specialised endoplasmic reticulum (ER)–mitochondria membrane contact sites [113]. Functionality of the VDAC1–stasimon interaction may be affected by reduced SMN levels, thereby implicating mitochondrial protein import in SMA pathology. Then rather curiously, recombinant SMN protein was shown to interact with TIM50 [114], a component of the TIM23 complex in the IMM that facilitates protein import from the TOM complex [115]. Within the TIM23 complex, TIM50 is the main receptor for inner membrane and matrix-bound proteins and is closely linked to ETC assembly. The physiological relevance of this interaction has not been examined further. Although it may be expected that cytoplasmic SMN interacts with mitochondrial TIM50 before mitochondrial import, putative localisation of SMN at the IMM in rat spinal cord [116] hints at the possibility that SMN may directly interact with TIM50 in mitochondria. These potential SMN–mitochondrial connections in protein import need further corroboration to enable understanding of their functional significance.

## SMN localisation at mitochondria

The prevailing viewpoint in the SMA field is that SMN does not localise to mitochondria [25, 117, 118]. However, re-evaluation of these studies suggests that there may be alternative interpretations of these conclusions. For example, although electron microscopy studies are technically challenging and their interpretation can be problematic, mitochondrial localisation of SMN in endogenous, disease-free rat spinal cord at multiple developmental stages was reported by Pagliardini et al. [116]. SMN was most convincingly detected in close apposition to the OMM, a location corroborated by colocalisation of SMN and ARX-2 at mitochondria in *C. elegans* [85]. Surprisingly, SMN was also reported at the IMM. Additional support for mitochondrial localisation of SMN, particularly at the OMM, can be gleaned from protein interactions: either directly through putative SMN interactions with mitochondrial proteins (Bcl-2, p53, and TIM50) or indirectly via SMN-interacting proteins that also localise to mitochondria, namely, ARX-2, stasimon, and neurocalcin delta (NCALD) [119], albeit most of these interactions have not been directly examined in this context. Transient localisation of SMN to mitochondria under certain cellular conditions and/or developmental stages seems likely. Given its function in transcription, translation, and proteostatic mechanisms, SMN is well suited to play a role in mitochondrial function. That mitochondria have recently been proposed as hubs of cellular proteostasis [120, 121] suggests this interaction could be reciprocal.

## A mitochondria-focused model of SMA disease and treatment

### The role of mitochondria in SMA

**Fig. 2:**
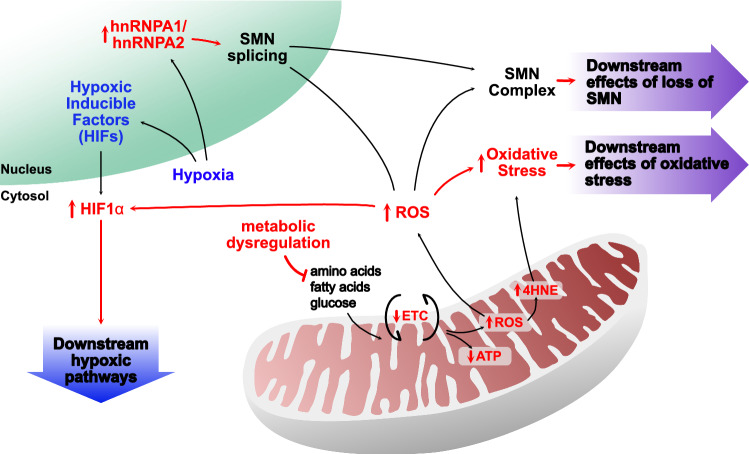
Energetic and metabolic changes in spinal muscular atrophy. Decreased activity in the electron transport chain (ETC) is found in patients with spinal muscular atrophy (SMA) and across models of SMA. This leads to a decrease in ATP and increased reactive oxygen species (ROS) production. ETC dysfunction may be linked to the metabolic dysregulation evident in SMA. Increased ROS production can modulate survival motor neuron (*SMN*) splicing as well as the SMN complex itself. Increased ROS causes oxidative stress, which is linked to an increase in 4-hydroxynoneal (4HNE) in SMA motor neurons. Hypoxia is another pathological process in SMA that also influences mitochondrial function, leading to an increase in production of hypoxic inducible factors (HIFs). In addition, HIF1α is modulated by ROS through ubiquitination processes involving SMN-relevant proteins, leading to activation of downstream hypoxic pathways. Hypoxia is also associated with an increase in *SMN* splicing factors, namely, heterogeneous nuclear ribonucleoprotein (hnRNP)-A1/A2

**Fig. 3:**
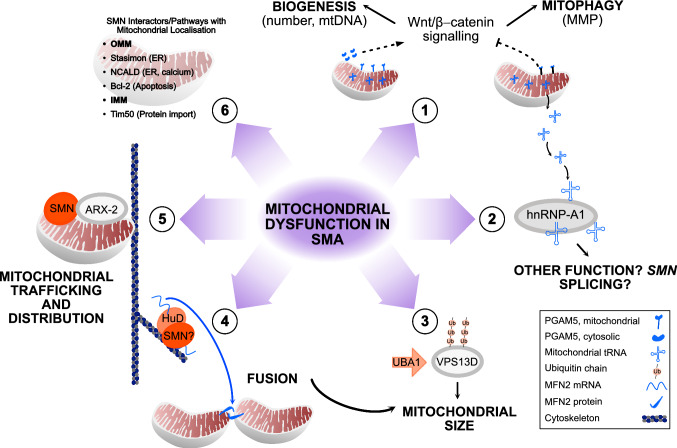
Mitochondrial dysfunction in spinal muscular atrophy. **1.** Following mitochondrial stress (e.g., hypoxia), mitochondrial PGAM family member 5 (PGAM5) inhibits Wnt/β-catenin signalling, leading to mitophagy (indirectly determined by mitochondrial membrane potential [MMP]). In addition, cytosolic PGAM activates Wnt/β-catenin signalling, leading to mitochondrial biogenesis (determined by mitochondrial number and mitochondrial DNA [mtDNA] production). This should ensure the removal of dysfunctional mitochondria and concurrent generation of new mitochondria to optimise the mitochondrial population during times of stress. The effect of survival motor neuron (SMN) in this mitochondrial stress pathway is not known but Wnt/β-catenin signalling is a known driver of spinal muscular atrophy (SMA) pathology. **2.** Mitochondrial transfer RNAs (tRNAs) are released from the mitochondrial matrix during mitochondrial stress, where they can interact with heterogeneous nuclear ribonucleoprotein (hnRNP)-A1 protein in the cytosol. The downstream effect of this interaction is not known but may involve mitonuclear communication or mitophagy. Alternatively, hypoxia is already known to alter *SMN* splicing via hnRNP proteins. **3.** Ubiquitination of vacuolar protein sorting 13 homolog D (VPS13D) by a ubiquitin-like modifier activating enzyme 1 (UBA1)-mediated mechanism influences mitochondrial size and morphology through fission and fusion mechanisms. **4.** HuD and SMN interact to transport mRNAs along the cytoskeleton. HuD has independently been shown to transport mRNA for the mitochondrial fusion protein, mitofusin 2 (MFN2), in pancreatic β cells (a known site of dysfunction in SMA). **5.** SMN colocalises with actin-related protein 2 (ARX-2) at mitochondria. Together both proteins are involved in mitochondrial trafficking and distribution. **6.** Other SMN interactors (Bcl-2, Tim50) and pathways (stasimon, NCALD) have mitochondrial locations, albeit the direct effect of these proteins and downstream functions (in brackets) has not been determined. Dashed lines indicate pathways that are linked to mitochondrial stress

Mitochondrial dysfunction has long been reported in patients with SMA. Unsurprisingly, mitochondrial energetic and metabolic alterations are associated with reduction of SMN protein and SMA pathology (Fig. 2). By combining current research, a model of mitochondrial dysfunction in SMA can be postulated that incorporates overlapping SMN functions (Fig. 3). Alterations in mitochondrial quality control mechanisms will lead to less efficient removal and reduced production of new mitochondria. Linking mitochondrial biogenesis and mitophagy to SMN-related function, β-catenin functions through the developmental Wnt signalling pathway [122] and was identified along with disrupted ubiquitin homeostasis as a driver of disease pathogenesis in SMA [50]. The cytosolic form of the mitochondrial phosphatase, PGAM family member 5 (PGAM5), was recently identified as a novel activator of Wnt/β-catenin signalling, stimulating mitochondrial biogenesis and increasing mitochondrial number in response to mitochondrial stress [123]. In addition, the mitochondrial form of PGAM5 acts as a repressor of Wnt/β-catenin signalling [124] and is involved in mitophagy [125, 126], again during times of mitochondrial stress. Opposing functions for related protein isoforms in the same signalling pathway suggests that Wnt/β-catenin signalling, and in turn SMN, may be important to mitochondrial turnover.

Altered mitochondrial quality control and trafficking/cytoskeletal defects may lead to changes in mitochondrial morphology, size, and subcellular distribution. Further mechanistic insights can again be inferred from known SMN-interacting proteins: HuD is an RNA-binding protein and SMN interactor [127], and together, both proteins influence mRNA transport [128]. Concurrently, HuD plays a role in mitochondrial dynamics, promoting mitochondrial fusion via transport of mitofusin 2 (*MFN2*) mRNA in mouse pancreatic β-cells [129], a known site of metabolic dysfunction in SMA. *MFN2* is an OMM protein that is essential for mitochondrial fusion, and also a causative gene for Charcot–Marie–Tooth disease [94]. Whether reduction of SMN levels in SMA motor neurons can affect HuD–MFN2 transport is not known but warrants further investigation, especially given that mitochondrial fusion appears impaired in SMA models. SMN also plays a well-described role in ubiquitin homeostasis with the ubiquitin protein, UBA1, a known SMN interactor that is already implicated in SMA pathology [130]. Of relevance here, this SMN-related pathway was recently linked to mitochondrial size mechanisms in *Drosophila* [131]. Specifically, vacuolar protein sorting 13 homolog D (Vps13D) was identified from a screen investigating ubiquitin and UBA1-mediated autophagy mechanisms during intestinal development. Vps13D was subsequently shown to play a role in mediating mitochondrial size through fission and fusion mechanisms. Potential overlapping of SMN and UBA1/ubiquitin function in this mitochondrial size pathway provides another point at which mitochondrial and SMN functions may intersect.

Mitochondrial structure is known to beget its function. Consequently, abnormalities in mitochondrial morphology and dynamics may be reflected in patients with SMA by reduced energetic capability at the cellular level and dysregulated metabolic control at the systems-wide level. Apoptosis should be inevitable but imbalances in SMN-related proteins (e.g., p53, Bcl-2 family) may hinder this process. Hypoxia could be an early influence on mitochondrial dysfunction, leading to increased mitochondrial ROS production and oxidative stress. Recently, hnRNPA1 (the *SMN2* splicing factor regulated by hypoxia) was shown to interact with a specific subset of mitochondrial transfer RNAs (tRNAs) [132] that were unexpectedly detected in the cytoplasm of HeLa cells. These proteins are typically found in the mitochondrial matrix where they are involved in translation of mitochondrial proteins. The biological significance of these cytosolic mitochondrial tRNAs is not yet apparent. They may be released following mitophagy and/or form part of regulatory crosstalk between mitochondria and the nucleus, possibly in times of mitochondrial stress. Further supporting this potential link between *SMN2* splicing and mitochondrial function, another hnRNP protein, hnRNPA2, also binds to *SMN2* exon 7 and alters SMN2 protein levels [133]. HnRNPA2 is an established transcriptional co-activator involved in mediating the nuclear response to mitochondrial stress, at least in mouse skeletal muscle cells [134]. Theoretically then, *SMN2* splicing may be altered by hnRNP proteins in response to mitochondrial stress resulting from hypoxia. Notably, the pattern of mitochondrial dysfunction described is not unique to SMA and is akin to that reported for other neurodegenerative diseases [135, 136]. However, layered throughout this model is the potential for involvement of SMN function, not least its roles in ubiquitination and autophagy. Understanding this underlying mitochondrial deficit and how it intersects with SMN function could benefit patients with SMA at all stages of disease. In this regard, it is also pertinent to ask whether insight into SMA disease mechanisms can be obtained from present understanding of mitochondria.

Mitochondria are unequivocally bioenergetic and biosynthetic organelles, yet current research suggests that they should not be viewed solely as such. Indeed, mitochondria are increasingly recognised as signalling hubs either through generation of second messengers (e.g., ROS, Ca^2+^, ATP and other metabolites) or by acting as scaffolds for signalling complexes that form on the OMM [137–139]. Likely linked to their signalling capacity, is the emerging role that mitochondria play in cell fate pathways aside from apoptosis, including the cell cycle [140–142] and cellular differentiation [143, 144]. Mitochondrial involvement in these cell fate pathways highlights an interplay between ROS signalling and mitochondrial dynamics, both of which are altered in SMA pathology. Indeed, effective execution of these cell fate roles is likely to be affected by a functionally suboptimal mitochondrial population. Future consideration of SMN function in mitochondrial signalling and cell fate pathways may reveal a role for mitochondria in SMA that lies outside their canonical energetic role.

### Mitochondria-targeted therapies for SMA

The primary treatment aimed at targeting mitochondria in SMA is Olesoxime, which made it through to phase III clinical trials. Olesoxime exerts its neuroprotective effects by modulating the mitochondrial permeability transition pore (an opening between the inner and outer mitochondrial membranes that is induced during pathology [145]), increasing cell survival in multiple in vitro and in vivo models [146]. Phase II clinical trials in non-ambulatory patients with Types 2/3 SMA showed a stabilisation, or even a slight increase, in motor function over the study period, whilst patients receiving a placebo showed a decline in motor function [147]. Unfortunately, Roche announced in 2018 that they were cancelling the phase III clinical trial due to difficulties in production and dosage as well as disappointing long-term results (https://www.treatsma.uk/wp-content/uploads/2018/05/2018-05-30-Olesoxime-PG-update-May-2018.pdf). However, since it is now clear that additional therapies are needed in combination with SMN replacement, cancelling Olesoxime clinical trials may come to be seen as premature and could still offer an option as a combinatorial (“SMN+”) therapy.

Targeting metabolic pathways is a second strategy that could benefit mitochondrial function in SMA. For example, targeting the glycolytic enzyme, phosphoglycerate kinase 1 (PGK1), either genetically or pharmacologically, can improve motor neuron health in SMA models [28]. Transcriptionally PGK1 is induced by HIF1α [148] and targeted to mitochondria following hypoxia [149], therefore the therapeutic benefit of PGK1 may involve hypoxic SMA mechanisms. Likewise, treatment with prednisolone (a corticosteroid that targets the transcription factor that modulates BCAA metabolism) or genetic overexpression of this transcription factor and therefore boosting of this metabolic pathway, doubled the lifespan of an intermediate mouse model of SMA. Glucocorticoids themselves are synthesised in mitochondria [150]. Potentially the therapeutic effects of prednisolone may also tap into mitochondrial-related glucocorticoid pathways.

More general metabolic approaches include changes to diet and exercise. In a mild SMA mouse model, forced exercise improved various markers of SMA pathology including fatiguability and motor neuron survival [151]. Further, a functional decrease in mitochondrial oxidative capacity was reversed by exercise intervention in a subset of muscle fibres in the same mouse model [31]. However, whether these results can be directly translated to human patients remains to be determined. A systematic review of evidence for and against exercise in patients with SMA was inconclusive due to heterogeneity of the patient groups and exercise performed [152]. This is obviously a difficult therapy to apply to patients with SMA due to their decreased motor abilities. A simpler target may be food intake. Targeting lipid dysregulation in SMA mice with a low-fat diet doubled their survival [58], whilst BCAA supplementation also improved SMA mouse model lifespan [57]. A recent systematic review described the lack of evidence for nutritional care in patients with SMA, an issue that is likely to become more pressing now that patients are living longer [153].

The early success of Olesoxime indicates that targeting mitochondria may still be an exciting avenue for future therapy development as a systemic, combinatorial option. Several therapies are being tested in other neurodegenerative diseases with mitochondrial dysfunction as a pathogenic hallmark, including metformin [154] and resveratrol [155] (see also recent reviews of mitochondrial therapies [156, 157]). Photobiomodulation is an alternative non-pharmacological approach that is gaining traction in neurodegenerative and brain injury fields [158, 159]. Photobiomodulation uses light energy to modulate biological function with reported mitochondrial effects including increased mitochondrial respiration and mtDNA levels [160], altered mitochondrial dynamics [161], increased MMP and ATP production [162, 163], and redox effects [164]. Given the demographic of the patient group, this is obviously an attractive strategy since it is non-invasive, has minimal side effects, and enables targeted, localised treatment. Of relevance to SMA, positive effects of photobiomodulation have been reported on muscle fatigue in healthy subjects [165], while there are indications of efficacy in models of Alzheimer’s disease, with improvements in both behavioural and molecular phenotypes [166]. Mitochondrial transplantation is another potential new avenue in mitochondrial therapies [167, 168], with the caveat that it is still very much at the experimental stage [169, 170]. This approach is based on the premise of transplanting entire mitochondria to treat disease tissue [171]. It has recently been shown that neurons can take up mitochondria from neighbouring astrocytes in stroke models [172], and this mechanism may be boosted by administering mesenchymal stem cells [173]. Given that multiple mitochondrial functions are disrupted in SMA, targeting mitochondria at the organelle level is appealing. These therapeutic approaches are yet to be tested in SMA models but highlight future research opportunities.

## Conclusions

As the genetic cause of SMA, SMN is imperative to disentangling the molecular phenotype of SMA and translating it back to the clinic. Yet determining the role of mitochondria in SMN function and SMA pathology may lead to clinical benefit that can complement SMN-replacement therapies. Mitochondria indisputably play an energetic role in motor neurons and muscle. Nevertheless, understanding of mitochondrial function has evolved considerably to show their more embedded role in wider cellular life. Intersection between SMN function and mitochondria can occur at multiple stages of SMA pathology. In turn, both SMN protein and mitochondria show pathological applicability that extends outside of motor neurons and muscle cells to affect every organ of the body. As SMA moves into a new era of treatment and disease progression, understanding the myriad roles of SMN protein and how they impact on generic and cell-type specific functions is ever more crucial for realisation of a cure for SMA.

## Data Availability

Not applicable.

## Abbreviations

ARX-2: Actin-related protein 2
BCAA: Branched-chain amino acid
Bcat2: BCAA transaminase 2
ETC: Electron transport chain
HIF: Hypoxia-inducible factor
4-HNE: 4-Hydroxynonenal
hnRNP: Heterogeneous nuclear ribonucleoprotein
IMM: Inner mitochondrial membrane
MFN2: Mitofusin 2
MMP: Mitochondrial membrane potential
mtDNA: Mitochondrial DNA
NAIP: Neuronal apoptosis inhibitory protein (or NLR family apoptosis inhibitor protein)
NCALD: Neurocalcin delta
OMM: Outer mitochondrial membrane
PGAM5: PGAM family member 5
PGK1: Phosphoglycerate kinase 1
ROS: Reactive oxygen species
SMA: Spinal muscular atrophy
SMN: Survival motor neuron
TIM: Translocase of the inner membrane
TOM: Translocase of the outer membrane
tRNA: Transfer RNA
UBA1: Ubiquitin-like modifier activating enzyme 1
VDAC1: Voltage-dependent anion-selective channel 1
Vps13D: Vacuolar protein sorting 13 homolog D
